# Machine Learning-Based Identification of Risk Factors for ICU Mortality in 8902 Critically Ill Patients with Pandemic Viral Infection

**DOI:** 10.3390/jcm14155383

**Published:** 2025-07-30

**Authors:** Elisabeth Papiol, Ricard Ferrer, Juan C. Ruiz-Rodríguez, Emili Díaz, Rafael Zaragoza, Marcio Borges-Sa, Julen Berrueta, Josep Gómez, María Bodí, Susana Sancho, Borja Suberviola, Sandra Trefler, Alejandro Rodríguez

**Affiliations:** 1 Intensive Care Department, Vall d’Hebron University Hospital, 08035 Barcelona, Spain; elisabeth.papiol@vallhebron.cat (E.P.); ricard.ferrer@vallhebron.cat (R.F.); juancarlos.ruiz@vallhebron.cat (J.C.R.-R.); 2 Shock, Organ Dysfunction and Resuscitation Research Group, Vall d’Hebron Research Institute (VHIR), 08035 Barcelona, Spain; 3 Medicine Department, Universitat Autònoma de Barcelona, 08035 Barcelona, Spain; 4 Critical Care Department, Hospital Parc Tauli, 08208 Sabadell, Spain; emilio.diaz.santos@gmail.com; 5 Critical Care Department, Hospital Dr. Peset, 46017 Valencia, Spain; zaragoza_raf@gva.es; 6 Critical Care Department, Hospital Son Llatzer, 07198 Palma de Mallorca, Spain; marcio.borges.sa1967@gmail.com; 7 Critical Care Department, Hospital Universitari Joan XXIII, 43005 Tarragona, Spain; juberrueta.hj23.ics@gencat.cat (J.B.); mbodi.hj23.ics@gencat.cat (M.B.); sitrefler@yahoo.es (S.T.); 8 Tarragona Health Data Research Working Group (TheDaR), 43005 Tarragona, Spain; 9 Technical Secretary Department, Hospital Universitari Joan XXIII, 43005 Tarragona, Spain; josep.goal@gmail.com; 10 Medicine Department, Rovira i Virgili University, 43002 Tarragona, Spain; 11 Pere Virgili Health Research Institute, 43005 Tarragona, Spain; 12 Centre for Biomedical Research Network Respiratory Diseases (CIBERES), 43005 Tarragona, Spain; 13 Critical Care Department, Hospital Universitario y Politécnico La Fe, 46026 Valencia, Spain; sancho_sus@gva.es; 14 Critical Care Department, Hospital Marques de Valdecilla, 39008 Santander, Spain; borjasuberviola1977@gmail.com; 15 Basic Sciences Department, Rovira I Virgili University, 43201 Tarragona, Spain

**Keywords:** ICU mortality, pandemic viruses, mortality risk factors, random forest, generalized linear model

## Abstract

**Background/Objectives**: The SARS-CoV-2 and influenza A (H1N1)pdm09 pandemics have resulted in high numbers of ICU admissions, with high mortality. Identifying risk factors for ICU mortality at the time of admission can help optimize clinical decision making. However, the risk factors identified may differ, depending on the type of analysis used. Our aim is to compare the risk factors and performance of a linear model (multivariable logistic regression, GLM) with a non-linear model (random forest, RF) in a large national cohort. **Methods**: A retrospective analysis was performed on a multicenter database including 8902 critically ill patients with influenza A (H1N1)pdm09 or COVID-19 admitted to 184 Spanish ICUs. Demographic, clinical, laboratory, and microbiological data from the first 24 h were used. Prediction models were built using GLM and RF. The performance of the GLM was evaluated by area under the ROC curve (AUC), precision, sensitivity, and specificity, while the RF by out-of-bag (OOB) error and accuracy. In addition, in the RF, the im-portance of the variables in terms of accuracy reduction (AR) and Gini index reduction (GI) was determined. **Results**: Overall mortality in the ICU was 25.8%. Model performance was similar, with AUC = 76% for GLM, and AUC = 75.6% for RF. GLM identified 17 independent risk factors, while RF identified 19 for AR and 23 for GI. Thirteen variables were found to be important in both models. Laboratory variables such as procalcitonin, white blood cells, lactate, or D-dimer levels were not significant in GLM but were significant in RF. On the contrary, acute kidney injury and the presence of *Acinetobacter* spp. were important variables in the GLM but not in the RF. **Conclusions**: Although the performance of linear and non-linear models was similar, different risk factors were determined, depending on the model used. This alerts clinicians to the limitations and usefulness of studies limited to a single type of model.

## 1. Introduction

Pandemics have historically been one of the greatest threats to public health, causing high mortality and exerting a significant impact on healthcare systems and society in general. Two recent pandemics have been particularly devastating: the influenza A (H1N1) virus, which emerged in 2009, and SARS-CoV-2, first identified in 2019. Both caused millions of deaths worldwide [1,2,3,4] and challenged the response capacities of healthcare systems, the pharmaceutical industry, and governments. They also exhibited ethical, economic, and social consequences that are still being felt today [5,6].

Despite the knowledge and advances in biomedicine, there are still limitations in the ability to predict the outcome of patients critically ill with pandemic viral infections. Early identification of patients at increased risk of mortality is essential to optimize intensive care unit (ICU) resources and improve clinical outcomes. Several authors [7,8,9,10,11] have identified a large number of risk factors associated with mortality in patients critically ill with influenza A (H1N1) and SARS-CoV-2 that differ or overlap, depending on the population studied, the country, or the method of analysis used. Traditionally, statistical models such as logistic regression have been used to quantify the association between confounding variables and the dependent variable in a linear fashion. However, this approach displays limitations in detecting non-linear relationships (perhaps the most common in medicine) and the complex interaction between multiple variables, which limits its predictive power in clinical scenarios with high-dimensional data [12].

In this context, new machine learning techniques have emerged as promising tools for predicting complex clinical outcomes. Among these, random forest, one of the most widely used techniques today, has shown significant advantages in identifying complex patterns in the data, without the need for parametric assumptions. This algorithm, based on the combination of multiple decision trees, offers greater predictive accuracy and robustness to the collinearity and heterogeneity of clinical data than those of linear models [13,14].

Our hypothesis is that different risk factors can be identified by applying different models of analysis. To test our hypothesis, the aim of our study is to identify risk factors associated with mortality in patients with severe pneumonia due to influenza A (H1N1) and SARS-CoV-2 infection by comparing the predictive ability of traditional logistic regression models with advanced machine learning techniques, specifically random forest. Our study aims to alert clinicians to the limitations of classical models and the need for more complex or multiple analyses to identify true risk factors and thus optimize decision making in the management of ICU patients.

## 2. Materials and Methods

### 2.1. Design

We conducted a secondary analysis of two prospective, multicenter cohort studies. The first dataset came from the GETGAG registry, a voluntary registry established by the Spanish Society of Intensive Care Medicine (SEMICYUC) in 2009 during the influenza A(H1N1)pdm09 pandemic. A total of 184 Spanish ICUs contributed data between June 2009 and June 2019 [15]. The Ethics Committee of Joan XXIII University Hospital (CEI no. 11809) and the ethics committees of all participating centers approved the study protocol. We did not obtain informed consent from patients because the study was observational, and all data were anonymized. The second dataset comes from the COVID-19 registry, a voluntary initiative created by SEMICYUC in 2020 during the SARS-CoV-2 pandemic. Seventy-four Spanish ICUs contributed data between 1 July 2020 and 31 December 2021 [15]. We retrospectively registered the study on ClinicalTrials.gov (NCT04948242) on 30 June 2021. The institution’s Internal Review Committee (Research Ethics Committee on Medicinal Products (CEIm) at the Pere Virgili Health Research Institute (IISPV), IRB# CEIM/066/2020) waived the requirement for informed consent. Local researchers maintained contact with the study team, and each participating hospital obtained approval from its local ethics committee. We conducted the study in accordance with the principles of the Declaration of Helsinki and the European Clinical Trials Directive 2001/20/EC on Good Clinical Practice [16].

We presented the results following the Strengthening the Reporting of Observational Studies in Epidemiology (STROBE) guidelines [17].

### 2.2. Study Population

We included a total of 8902 consecutive patients who required ICU admission due to respiratory infections caused by influenza A (H1N1)pdm09, seasonal influenza A or B (n = 3702), or SARS-CoV-2 (n = 5200) during the respective study periods. We confirmed the presence of each virus by performing real-time polymerase chain reaction (rt-PCR) in each hospital, according to Infectious Diseases Society of America (IDSA) recommendations for influenza [18] and World Health Organization (WHO) recommendations for SARS CoV-2 [19]. We monitored each patient until confirmed ICU discharge or death, whichever occurred first.

### 2.3. Definitions

We considered co-infection in patients who presented with lower respiratory tract infection symptoms and radiographic evidence of pulmonary infiltrates unexplained by other causes [20]. We confirmed coinfection through laboratory testing based on the criteria established by the Centers for Disease Control and Prevention (CDC) [20,21]. Only respiratory infection microbiologically confirmed with a respiratory specimen or serology obtained within 2 days of ICU admission was considered community-acquired coinfection. The diagnosis of coinfection was considered “definitive” if respiratory pathogens were isolated from blood or pleural fluid and if serological tests confirmed a four-fold increase of atypical pathogens, including *Chlamydia* spp., *Coxiella burnetti*, and *Moraxella catarrhalis*. Only patients with confirmed microbiologic diagnosis were included in the present analysis.

We diagnosed acute kidney injury (AKI) based on the Acute Kidney Injury Network (AKIN) criteria, as defined in the international KDIGO guidelines [22].

We defined appropriate empiric antibiotic treatment (AEAT) as the administration of antibiotics at ICU admission before microbiological results were available, followed by adjustment according to pathogen susceptibility once results became known. The attending physician at each center determined whether treatment met these criteria.

We defined inappropriate empiric antibiotic treatment (IEAT) as antibiotic therapy started at ICU admission that was not adjusted to the pathogen’s susceptibility once microbiological results became available. This definition also included the administration of antibiotics to patients without documented bacterial co-infection.

We defined GAP-UCI as the time elapsed between the diagnosis of the pandemic viral infection and ICU admission.

We defined GAP-Diagnosis as the time between the onset of clinical symptoms and the microbiological confirmation of the pandemic viral infection.

### 2.4. Study Variables

We collected demographic data, comorbidities, and clinical and laboratory findings within the first 24 h after ICU admission. We also recorded whether patients required invasive mechanical ventilation and whether they presented with shock upon arrival. We assessed disease severity using the Acute Physiology and Chronic Health Evaluation II (APACHE II) score [23] and the level of organ dysfunction using the SOFA score [24]. The variables included in the study are detailed in Table 1.

### 2.5. Missing Data Management

We excluded continuous variables with more than 30% missing data from the database. For variables with fewer missing values, we applied imputation using the missForest package in R/CRAN. This method was used to impute missing values for D-dimer (18%), lactate dehydrogenase (15%), procalcitonin (15%), creatinine (14%), SOFA score (14%), APACHE II score (10%), and C-reactive protein (5%). Categorical data, including ICU mortality, were complete for all patients.

### 2.6. Analysis Plan and Statistical Analysis

Firstly, we calculated the crude ICU mortality rate for the overall population and compared patient characteristics based on outcomes. We expressed qualitative variables as percentages and summarized quantitative variables as medians with interquartile ranges (Q1–Q3). To assess differences between groups, we applied the Chi-square and Fisher’s exact tests for categorical variables and the Student’s *t*-test or Mann–Whitney U-test for quantitative variables.

Secondly, we applied a binary logistic regression model to identify variables independently associated with all-cause ICU mortality. We incorporated into the generalized linear model (GLM) all variables that were statistically significant (*p* < 0.05) in the bivariate analyses. We developed the mortality prediction model using only variables available at the time of ICU admission. To improve model performance, we categorized continuous variables, defining cut-off points based on the median values observed in surviving patients. We expressed the results as odds ratios (OR) with 95% confidence intervals.

To validate the model internally, we randomly divided the population into a development set (training data) containing 70% of patients and a validation set (testing data) with the remaining 30%. We assessed model performance by calculating accuracy, precision, sensitivity, specificity, and the area under the ROC curve (AUC). We also examined collinearity among explanatory variables using variance inflation factors (VIF).

In addition, we performed k-fold cross-validation with k = 10. This approach involved splitting the original dataset into a training set and a validation set. We further divided the training data into ten subsets. Each subset served once as the test set, while the remaining nine subsets were used for model training. After completing all iterations, we calculated accuracy and error for each model. We then averaged these results across the ten folds to obtain the final accuracy and error estimates.

Thirdly, because of the significant imbalance between groups, e.g., only 25% of patients belonged to the deceased group, we considered that this class discrepancy could affect the model’s performance in predicting mortality. To test whether class imbalance influenced the linear model’s results, we applied the ROSE (random over-sampling examples) package. This statistical package generates balanced samples through a smoothed bootstrap approach, enabling reliable estimates of classifier accuracy when the minority class is rare. ROSE also provides traditional methods to address class imbalance and includes multiple metrics for assessing accuracy, which can be estimated via cross-validation, bootstrapping, or the holdout method [25,26]. We implemented the under option, which subsamples the majority class without replacement until either the specified sample size (N) is reached or the positive examples achieve a predefined probability (p). This method reduces the resulting sample size. We used the ROSE software (version 0.0-4) exclusively on the training subset, leaving the test subset unchanged. After developing the model on the training data, we applied it to the test set and evaluated its performance. We reported results as odds ratios (OR) with 95% confidence intervals, along with accuracy, sensitivity, specificity, and the area under the ROC curve (AUC).

Fourthly, to test our hypothesis, we developed a non-linear model using a random forest classifier (RFc). This technique is a powerful, tree-based machine learning approach. We configured our model to generate 500 random trees, each considering at least 15 variables. We evaluated model performance by calculating the out-of-bag (OOB) error, which estimates prediction error through bootstrap aggregation. Additionally, we assessed variable importance by examining the average loss of accuracy and the Gini index. The Gini index, reported as “MeanDecreaseGini”, measures the degree of disorder: higher values indicate greater importance in the model because scores near 0 imply higher disorder, while those closer to 1 reflect lower disorder and more consistent contribution to the outcome. For internal validation, we randomly split the population into a training set (70% of patients) and a test set (30%). We determined model performance by measuring accuracy.

We performed all statistical analyses using R statistical software (version 4.4.1) from The R Project for Statistical Computing (r-project.org).

## 3. Results

### 3.1. Whole Population

We included a total of 8902 ICU patients in the study: 3702 (41.6%) were diagnosed with influenza, and 5200 (58.4%) with coronavirus disease 2019 (COVID-19). All diagnoses were confirmed by polymerase chain reaction (PCR). Table 1 shows the general characteristics of patients by ICU outcome. The cohort was predominantly male (65.8%), with a mean age of 60 years. Disease severity was moderate, with mean APACHE II and SOFA scores of 14 and 5, respectively. The most common comorbidities were obesity, diabetes, and chronic obstructive pulmonary disease (COPD). The mean ICU stay was 13 days, and the crude ICU mortality rate reached 25.8%. Compared to survivors, non-survivors were older and exhibited more severe illness, greater systemic inflammation, more comorbidities, higher requirements for organ support, and longer ICU stays. Coinfection was also more frequent among non-survivors, with significant differences observed in pathogens such as *Pseudomonas aeruginosa*, *Aspergillus* spp., and *Acinetobacter* spp. (Table 1).

### 3.2. Factors Associated with Crude ICU Mortality According to General Linear Model (GLM)

**Figure 1:**
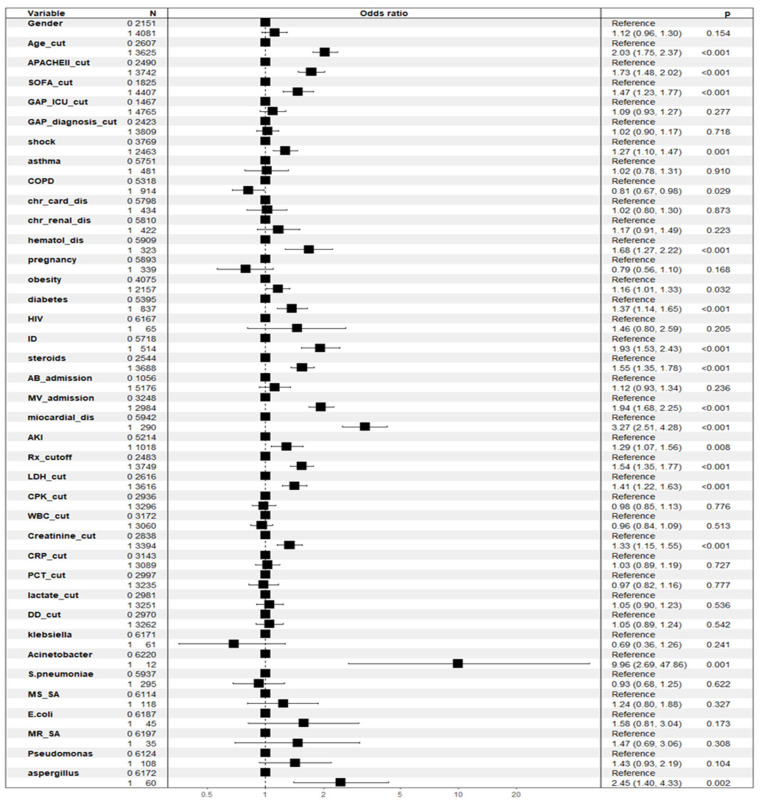
Odds ratio (OR) plot of variables associated with ICU crude mortality in linear multivariate analysis (GLM). Abbreviations: cut: cut-off; APACHE II: Acute Physiology and Chronic Health Evaluation; SOFA: sequential organ failure assessment; AB: antibiotics; CPK: creatine phosphokinase; DD: D-dimer; MR_SA: methicillin-resistant *S. aureus*; MV: invasive mechanical ventilation; WBC: white blood cells; COPD: chronic obstructive pulmonary disease; dis: disfunction; Chr_Card_dis; chronic cardiac disease; HIV: human immunodeficiency virus; AKI: acute kidney injury; CRP: C-reactive protein; GAP_ICU_cut: time elapsed between diagnosing pandemic viral infection and admission to ICU; Chr_renal_dis: chronic renal disease; ID: immunosuppression; Rx-cutoff: > 2 fields with infiltrations in chest X-ray; PCT: procalcitonin; MS_SA: methicillin-sensitive *S. aureus*; GAP_diagnsosis_cut: time from symptoms onset to diagnosis; hematol_dis: hematologic disease; LDH: lactate dehydrogenase.

We used multiple logistic regression to examine the associations between crude ICU mortality (the dependent variable) and various independent variables. The model included the following factors: sex (male), age cut-off, APACHE II cut-off, SOFA cut-off, ICU GAP cut-off, GAP diagnosis cut-off, shock, asthma, COPD, chronic heart disease, chronic kidney disease, hematological disease, pregnancy, obesity, diabetes, HIV, immunosuppression, steroid use, and antibiotic treatment at ICU admission. Additional variables included mechanical ventilation at ICU admission, myocardial dysfunction, acute kidney injury (AKI), more than two areas of infiltration on chest X-ray, lactate dehydrogenase (LDH) cut-off, creatine phosphokinase (CPK) cut-off, white blood cell (WBC) cut-off; C-reactive protein (CRP) cut-off; procalcitonin (PCT) cut-off; lactate cut-off; D-dimer (DD) cut-off; presence of *Klebsiella* spp., *Acinetobacter* spp., *Streptococcus pneumoniae*, methicillin-sensitive *Staphylococcus aureus* (MSSA), *E. coli*, methicillin-resistant *S. aureus* (MRSA), *Pseudomonas aeruginosa*, and *Aspergillus* spp.; and administration of an antiviral vaccine. Among these, 17 variables were independently associated with all-cause ICU mortality. The significant factors are detailed in Figure 1 and Table 2.

### 3.3. Linear Model (GLM) Validation

When we applied the developed model to the test subset, it performed acceptably, achieving an accuracy of 76%, a sensitivity of 61%, and a specificity of 79% (see Supplementary Table S1). The area under the curve (AUC) was 0.76 (95% CI, 0.74–0.78; see Supplementary Figure S1). We did not detect collinearity among the included variables (see Supplementary Table S2). Cross-validation with k = 10 did not improve overall accuracy (which remained at 76%) but increased sensitivity to 94% while reducing specificity to 26% (see Supplementary Table S3).

### 3.4. Development of the GLM Model with Correction of Class Imbalance

When we applied the ROSE package to the training set, the number of patients decreased from 6232 to 3152. Among these, 1606 died, resulting in an estimated mortality rate of 50.9%, which was double the actual rate of 25%. Developing the linear GLM model with this balanced dataset did not improve performance, yielding an AUC-ROC of 76% (95% CI, 74–78%) and an accuracy of 68%. Supplementary Figures S2 and S3 and Table S4 provide details of the model development. Because this approach did not optimize results and reduced the sample size substantially, we decided to retain the original GLM model despite the class imbalance, as it did not appear to affect performance.

### 3.5. Factors Associated with ICU Mortality According to No-Linear Model (Random Forest)

We developed a random forest classifier (RFc) model to analyze the impact of confounding variables on ICU mortality in a non-linear manner. To enable comparison, we included the same independent variables used in the GLM. The RFc model yielded an out-of-bag (OOB) error rate of 25.3%.

**Figure 2:**
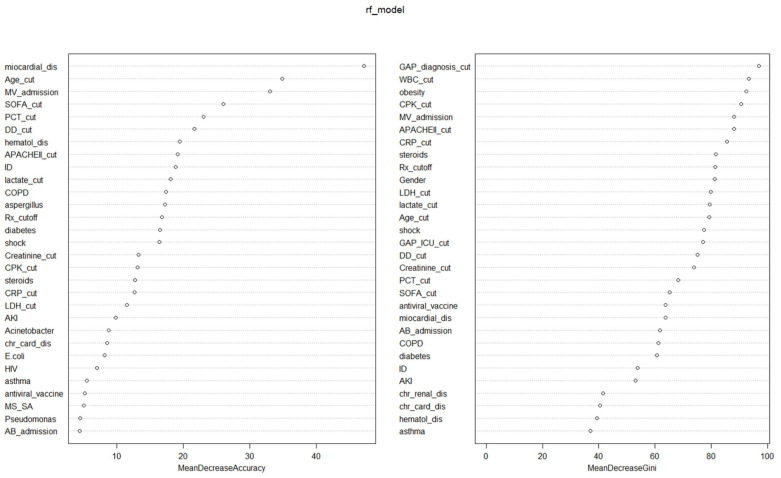
Contribution of each confounding variable according to the random forest (RF) model for variables associated with all-cause ICU mortality. Abbreviations: cut: cut-off; APACHE II: Acute Physiology and Chronic Health Evaluation; SOFA: sequential organ failure assessment; AB: antibiotics; CPK: creatine phosphokinase; DD: D-dimer; MR_SA: methicillin-resistant *S. aureus*; MV: invasive mechanical ventilation; WBC: white blood cells; COPD: chronic obstructive pulmonary disease; dis: disfunction; Chr_Card_dis; chronic cardiac disease; HIV: human immunodeficiency virus; AKI: acute kidney injury; CRP:C-reactive protein; GAP_ICU_cut: time elapsed between diagnosing pandemic viral infection and admission to ICU; Chr_renal_dis: chronic renal disease; ID: immunosuppression; Rx-cutoff: > 2 fields with infiltrations in chest X-ray; PCT: procalcitonin; MS_SA: methicillin-sensitive *S. aureus*; GAP_diagnsosis_cut: time from symptoms onset to diagnosis; hematol_dis: hematologic disease; LDH: lactate dehydrogenase).

Nineteen variables reduced model precision by more than 10% (Table 2 and Figure 2). Notably, obesity, acute kidney injury (AKI), and the presence of *Acinetobacter* spp. were important predictors in the GLM but did not contribute significantly to precision in the RFc model. In contrast, COPD, lactate, procalcitonin, D-dimer, and CPK were relevant for accuracy in the RFc model but not in the GLM.

Additionally, twenty-three variables were associated with a reduction in Gini greater than 50% in the non-linear analysis. AKI, *Acinetobacter* spp. and *Aspergillus* spp. were significant in the GLM but not relevant to Gini reduction. Conversely, GAP diagnosis, GAP ICU, male sex, and WBC count were important contributors to Gini decrease in the RF model (see Table 2 and Figure 2).

### 3.6. Non-Linear Model (RFc) Validation

We applied the developed model to the test subset, where it achieved an acceptable accuracy of 75.6%. This performance closely matched that of the linear GLM model, despite differences in the covariates used.

### 3.7. Patient Classification by Model

**Figure 3:**
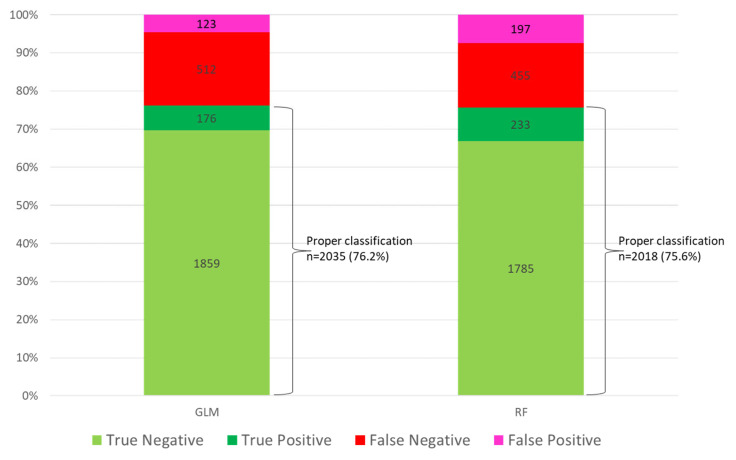
Classification of patients according to the linear (generalized linear model—GLM) and non-linear (random forest—RF) models.

**Figure 4:**
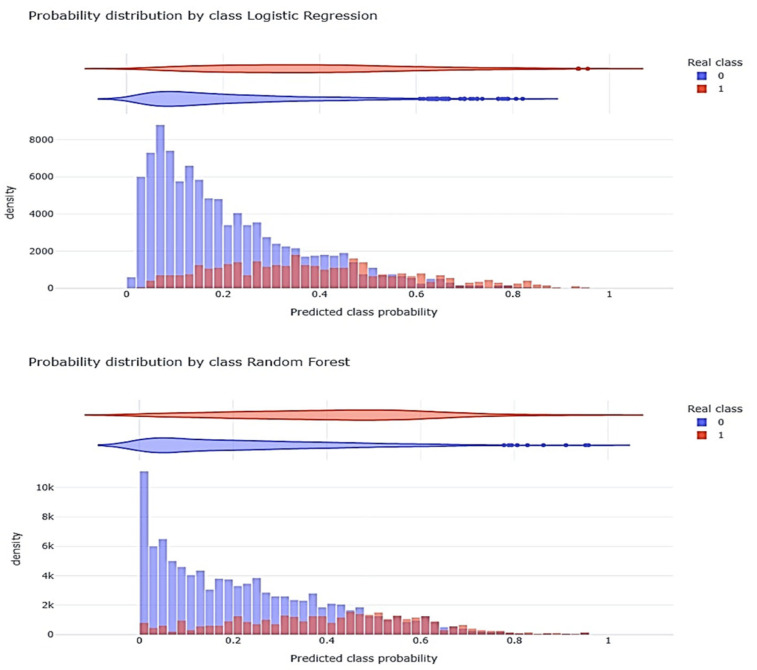
Distribution of the probability generated by each model (Class) with respect to the observed results (Real); (0 = survivors; 1 = non-survivors).

Of the 2670 patients in the test set, the GLM correctly classified 2035 (76.2%), and the random forest (RF) correctly classified 2018 (75.6%) (see Figure 3 and Supplementary Figure S4A,B). Both models agreed on the classification of 1872 patients (70.1%), and 489 patients were misclassified (18.3%). Figure 4 illustrates the probability distributions generated by each model (Class) compared to the actual outcomes (Real).

## 4. Discussion

To the best of our knowledge, this is the first study to use machine learning techniques for a large number of critically ill patients affected by pandemic viruses. Our main finding was that generating mortality prediction models using either a linear technique (GLM) or a non-linear technique (RF) was associated with similar performance, with an accuracy close to 80%.

However, the risk factors identified differed according to the type of analysis used. While factors such as age, severity, degree of organ dysfunction, and need for mechanical ventilation were important in both models (major determinants), other laboratory variables such as procalcitonin, D-dimer, and lactate levels were only identified in the RF model (minor determinants). Conversely, acute kidney injury (AKI) and the presence of *Acinetobacter* spp. were significant only in the GLM (minor determinants). These findings should alert clinicians to the limitations and implications of studies that rely exclusively on one methodological approach to identify prognostic factors.

The influenza A (H1N1) and SARS-CoV-2 pandemics have put enormous pressure on healthcare systems around the world, highlighting the urgent need for reliable and accurate methods to predict patient outcomes in order to manage resources appropriately. Although pandemics may seem to be a thing of the past, each winter, hospitals are overwhelmed by patients with respiratory failure due to viral infections, generating seasonal surges in ICU admissions and demand for resources.

The early identification of high-risk patients with viral infections is essential. It allows for rapid triage, targeted intensive care, and optimized resource allocation, all of which can ultimately improve patient outcomes. Against this backdrop, our study sought to evaluate and compare the performance of traditional statistical and machine learning models in predicting mortality, as well as exploring how each method identifies different clinical predictors of outcome.

Several authors have used different types of machine learning (ML) analysis to identify risk factors and develop predictive models for patients with SARS-CoV-2, while we did not find any studies involving influenza A H1N1. Additionally, most studies included hospitalized patients, with few critically ill patients. Huang et al. [27] reported an AUC of 94.4%, a sensitivity of 94.1%, and a specificity of 90.2% when using ML, but the population considered comprised only 127 patients, of whom 33 were critically ill. Meanwhile, Zhu et al. [28] examined 127 patients with confirmed cases of SARS-CoV-2 (16 of whom were severely ill), Gong et al. [29] examined 372 patients with confirmed cases of SARS-CoV-2 who were hospitalized, Aloisio et al. [30] examined 427 patients with confirmed cases of SARS-CoV-2, and Liu et al. [31] examined 336 severely ill patients with confirmed cases of SARS-CoV-2 (34 of whom died). All of these studies showed excellent performance (AUC > 90%) using linear logistic regression models. The small number of patients included in these studies limits the strength and generalizability of the results.

In line with our research, Reina-Reina et al. [32] conducted a sophisticated study on a population of 1200 patients with confirmed cases of SARS-CoV-2. The study assessed the risk of death and ICU admission using various machine learning (ML) techniques, including support vector machine (SVM), logistic regression (LR), k-nearest neighbors, decision tree, Gaussian naive Bayes, multi-layer perceptron (MLP), and ensemble methods such as AdaBoost and bagging. The authors found no significant differences in classification accuracy (>88%) between the different ML techniques. However, they opted for logistic regression (LR) as the algorithm for optimization due to the interpretability of the model, which is crucial in the medical field, despite random forest (RF) achieving slightly better average results. The model identified the most important variables as COPD, which increases the probability of death by 575%; age, which increases the probability by 145% every 10 years; and acute respiratory failure, which increases the probability by 513%. However, the authors do not report the differences between the predictor variables identified by each model, and only a small percentage of patients were critical.

Pourhomayoun et al. [33] applied various machine learning (ML) models (support vector machine (SVM), neural networks (NN), random forest (RF), decision tree, and logistic regression (LR)) to predict severity in a large cohort of more than 2,670,000 patients with SARS-CoV-2 infection. The original dataset contained 32 data points for each patient, including demographic and physiological data. The NN algorithm achieved the best performance and accuracy, with an area under the curve (AUC) of 89.98%, compared to 87.93% for random forest (RF) and 87.91% for logistic regression (LR). However, the authors did not conduct a statistical comparison of the AUCs to determine significance, nor did they compare the predictive factors of the different models, only presenting the NN factors in the form of a heat map. Furthermore, the severity of the patients’ disease was not reported.

In an excellent review of machine learning (ML) techniques used for prognosis in patients with SARS-CoV-2 infection, Alballa et al. [34] note that the most commonly used algorithm for diagnostic and prognostic models is logistic regression (LR), followed by XGBoost and finally, support vector machine (SVM). The authors point out that most of the studies included in the review used unbalanced datasets. In these studies, the majority of records in the training dataset represent the negative class (survivors), while the positive class (non-survivors) is under-represented. Consequently, the performance of various ML algorithms applied in the context of COVID-19 may be biased. In such cases, a high accuracy score could be attributed to the model’s ability to accurately identify negative samples and erroneously exclude all positive cases. In our study, we recognized and addressed this bias by applying subsampling to the majority class. However, this did not improve the performance of the balanced model compared to that of the unbalanced model, showing that class imbalance does not affect model reliability. This may be because the mortality rate among our critically ill patients is 25%, whereas in most published studies, it is around 10–15% [8,32,35] due to the absence of critically ill patients.

As our study revealed, the linear model performs similarly to non-linear models when it comes to predicting mortality in patients with COVID-19, a finding that has been corroborated by several other studies [32,33,34,35]. However, despite the structural flexibility of machine learning models for predicting outcomes in patients with this disease, there are limitations to their practical use. These include high heterogeneity between patients’ clinical profiles and small sample sizes, which may reduce the external validity and generalizability of the data. Most studies describe different risk factors and performances depending on which factors are included. This is consistent with studies [32,35,36,37] reporting the modest performance of machine learning (ML) models when trained exclusively with baseline clinical data collected at the time of intensive care unit (ICU) admission. The most successful predictive models, such as those of Wang et al. [38] and Karasneh et al. [39], incorporate dynamic, therapeutic, or immunological variables that significantly improve model performance. However, these variables are not available during the initial hours of care for critically ill patients, limiting their applicability to clinical practice.

We would like to highlight the strengths of our study. Firstly, the large number of critically ill patients included (n = 8902), of whom more than 3000 were affected by influenza A (H1N1)pdm09, makes it unique in its results. As it is a national multicenter study involving more than 148 ICUs in Spain, its results can be generalized to the whole country. Furthermore, it reports not only on the performance of the developed models, but also on the different risk factors identified and how patients are classified by each model. Based on these findings, we can classify risk factors as either major or minor determinants, depending on whether they are important in both models or only one. Recognizing these risk factors could be valuable in clinical practice for determining the prognosis of critically ill patients with a pandemic virus infection. Finally, our study alerts clinicians to the limitations of using models developed using a single method of analysis.

However, our study reflects limitations that need to be recognized. Firstly, despite the large number of patients and the study’s multicenter nature, these findings cannot be extrapolated to other populations (non-critical), health systems, or continents without local validation. Secondly, while the potential bias due to class imbalance has been addressed, other biases cannot be ruled out, such as those related to ethnicity or other confounding variables, as these variables are not included in our data. Thirdly, including data on ICU evolution in the models could potentially improve performance. However, our aim was to identify early risk factors for mortality that could be modified by clinicians to improve prognosis.

## 5. Conclusions

Our study highlights the continued relevance of linear models (GLM) for predicting mortality in the era of machine learning analysis. However, it alerts clinicians to the need for a complementary approach combining linear and non-linear analysis in order to identify all the major and minor determinants of mortality, with the ultimate goal of improving the prognosis of this critical patient group.

## Figures and Tables

**Table 1:** Baseline characteristics of the 8902 patients included in the analysis, categorized by ICU outcome and variable cut-off.

Variable	Whole Population (n = 8902)	Survival (n = 6608)	Non-Survival (n = 2294)	*p*-Value
General				
Age, median (Q1–Q3) years	60 (49–70)	58 (48–68)	67 (57–74)	<0.001
Age cut-off > 58 years, n (%)	5177(58.1)	3473 (52.6)	1704 (74.3)	<0.001
Male sex, n (%)	5855 (65.8)	4248 (64,3)	1607 (70.1)	<0.001
APACHE II, median (Q1–Q3)	14 (10–19)	13 (10–17)	17 (13–22)	<0.001
APACHE II cut-off > 13, n (%)	5309 (59.6)	3536 (53.5)	1773 (77.3)	<0.001
SOFA score, median (Q1–Q3)	5 (3–7)	4(3–7)	6(4–9)	<0.001
SOFA cut-off > 4, n (%)	6274 (70.5)	4299 (65.1)	1975 (86.1)	<0.001
GAP UCI, median (Q1–Q3)	1 (1–3)	1 (1–3)	2 (0–4)	<0.001
GAP UCI cut-off > 1 day, n (%)	6804 (76.4)	5085 (77.0)	1719 (74.9)	0.053
GAP diagnosis, median (Q1–Q3)	4 (1–7)	3 (1–7)	4 (1–7)	0.012
GAP diagnosis cut-off > 3 days, n (%)	5413 (60.8)	3943 (59.7)	1470 (64.1)	<0.001
> 2 fields with infiltrations in chest X-ray, n (%)	5343 (60.0)	3775 (57.1)	1568 (68.4)	<0.001
Antiviral vaccine, n (%)	1333 (14.9)	885 (13.4)	448 (19.5)	<0.001
Shock at ICU admission, n (%)	3549 (39.9)	2286 (34.6)	1263 (55.1)	<0.001
Laboratory				
White blood cells count, median (Q1–Q3) × 10^3^	8.6 (5.7–12.5)	8.5 (5.7–12.1)	9.0 (5.8–13.7)	<0.001
White blood cells count cut-off < 8.5 × 10^3^, n (%)	4405 (49.5)	3351 (50.7)	1054 (45.9)	<0.001
Lactate dehydrogenase, median (Q1–Q3) U/L	542 (403–687)	524 (378–665)	590 (458–749)	<0.001
Lactate dehydrogenase cut-off > 500 U/L, n (%)	5157 (57.9)	3593 (54.4)	1564 (68.2)	<0.001
C-reactive protein, median (Q1–Q3) mg/dL	19.6 (9.8–34.7)	19.0(9.5–34.4)	21.1 (10.4–35.4)	0.001
C-reactive protein cut-off >20 mg/dL, n (%)	4387 (49.3)	3184 (48.2)	1203 (52.4)	<0.001
Procalcitonin, median (Q1–Q3) ng/mL	0.88 (0.20–5.67)	0.83 (0.20–5.08)	1.04 (0.23–8.20)	<0.001
Procalcitonin cut-off >0.80 ng/mL, n (%)	4606 (51.7)	3350 (50.7)	1256 (54.8)	0.001
Lactate, median (Q1–Q3) mmol/L	2.0 (1.4–3.3)	2.0 (1.3–3.2)	2.2 (1.4–3.8)	<0.001
Lactate cut-off > 2 mmol/L, n (%)	4660 (52.3)	3369 (51.0)	1291 (56.3)	<0.001
Creatinine, median (Q1–Q3) mg/dL	0.89 (0.7–1.2)	0.85 (0.68–1.12)	1.01 (0.75–1.50)	<0.001
Creatinine cut-off >0.85 mg/dL, n (%)	4841 (54.4)	3330 (50.4)	1511 (65.9)	<0.001
D-dimer, median (Q1–Q3) ng/mL	3071 (971–6604)	2716 (900–6000)	4180 (1200–8680)	<0.001
D-dimer cut-off > 2700 ng/mL, n (%)	4663 (52.4)	3314 (50.2)	1349 (58.8)	<0.001
creatine phosphokinase, median (Q1–Q3) U/L	216 (100–420)	210 (97–414)	234 (111–442)	0.001
Creatine phosphokinase cut-off > 200 U/L, n (%)	4707 (52.9)	3433 (52.0)	1274 (55.5)	0.003
Comorbidities				
Diabetes mellitus, n (%)	1196 (13.4)	756 (11.4)	440 (19.2)	<0.001
Asthma, n (%)	698 (7.7)	556 (8.4)	142 (6.2)	0.001
COPD, n (%)	1281 (14.4)	936 (14.2)	345 (15.0)	0.32
Chronic heart disease, n (%)	623 (7.0)	418 (6.3)	205 (8.9)	<0.001
Chronic liver disease, n (%)	595 (6.7)	357 (5.4)	238 (10.4)	<0.001
Pregnancy, n (%)	480 (5.4)	399 (6.0)	81 (3.5)	<0.001
Obesity, n (%)	3046 (34.2)	2256 (34.1)	790 (34.4)	0.81
Human immunodeficiency virus, n (%)	144 (1.6)	107 (1.6)	37 (1.6)	1.00
Hematologic disease, n (%)	436 (4.8)	237 (3.6)	199 (8.7)	<0.001
Immunosuppression, n (%)	711 (8.0)	401 (6.0)	310 (13.5)	<0.001
Treatment				
Steroids, n (%)	5275 (59.2)	3746 (56.7)	1529 (66.7)	<0.001
Antibiotics (AB) at ICU admission, n (%)	7410 (83.2)	5428 (82.1)	1982 (86.4)	<0.001
Appropriate empiric AB treatment, n (%)	951 ((10.7)	671 (10.2)	280 (12.2)	0.007
High flow nasal cannula at admission, n (%)	1438 (16.1)	1138 (17.2)	300 (13.1)	<0.001
Invasive mechanical ventilation, n (%)	4252 (47.8)	2751 (41.6)	1501 (65.4)	<0.001
Most common aetiology of coinfection				
Coinfection, n (%)	1211 (100)	810 (12.3)	401 (17.5)	<0.001
Methicillin-sensitive *S. aureus* (MSSA), n (%)	172 (14.2)	111 (13.7)	61 (15.2)	0.47
*Pseudomonas aeruginosa*, n (%)	143 (11.8)	82 (10.1)	61 (15.2)	0.01
*Klebsiella* spp. N (%)	85 (7.0)	60 (7.4)	25 (6.2))	0.45
*Aspergillus* spp., n (%)	78 (6.5)	33 (4.0)	45 (11.2)	<0.001
*E. coli*, n (%)	69 (5.7)	43 (5.3)	26 (6.3)	0.40
Methicillin-resistant *S. aureus* (MRSA). n (%)	56 (4.6)	33 (4.0)	23 (5.7)	0.19
*Acinetobacter* spp., n (%)	17 (1.4)	4 (0.5)	13 (3.2)	<0.001
Outcomes				
ICU LOS, median (Q1–Q3) days	13 (6–23)	12 (6–23)	14 (7–24)	0.03
Acute kidney injury, n (%)	1435 (16.1)	855 (12.9)	580 (25.3)	<0.001

APACHE II: Acute Physiology and Chronic Health Evaluation; SOFA: sequential organ failure assessment; GAP-UCI: time from diagnosis to ICU admission; GAP-Diagnosis: time from symptoms onset to diagnosis; ICU: intensive care unit; LOS: length of stay.

**Table 2:** Variables associated with ICU mortality in the linear multivariate analysis (GLM) and non-linear multivariate analysis (random forest). Significant variables in the linear model and those with a significance greater than 10% for the decrease in accuracy or greater than 50% for the decrease in Gini in the non-linear model are shown.

	GLM Model		Random Forest Model	
Variable	OR	95%CI	Decreased Accuracy	Decreased Gini
Age ≥ 58 years	2.03	1.74–2.36	34.9%	79.2%
APACHE II ≥ 13 points	1.72	1.48–2.02	19.1%	88.1%
SOFA ≥ 4 points	1.47	1.23–1.76	26.0%	65.1%
Shock	1.27	1.09–1.47	16.4%	77.4%
Hematologic disease	1.67	1.26–2.22	19.5%	39.4%
Obesity	1.16	1.01–1.32	-----	92.4%
Diabetes	1.37	1.14–1.65	16.5%	60.6%
Immunosuppression	1.92	1.53–2.42	18.9%	53.0%
Steroids	1.54	1.34–1.77	12.7%	81.6%
Mechanical ventilation	1.94	1.67–2.25	33.0%	88.1%
Myocardial dysfunction	3.27	2.53–4.28	47.2%	63.6%
Acute kidney injury	1.29	1.07–1.55	----	-----
>2 fields with infiltrations in chest X-ray	1.54	1.34–1.77	16.8%	81.3%
LDH ≥ 500 U/L	1.41	1.22–1.63	11.5%	79.7%
Creatinine ≥ 0.85 mg/dL	1.33	1.14–1.55	13.3%	73.8%
*Acinetobacter* spp.	9.95	2.61–47.8	----	----
*Aspergillus* spp.	2.45	1.39–4.33	11.2%	----
Procalcitonin ≥2 ng/mL	----	----	23.0%	68.1%
D-dimer ≥ 2700 ng/mL	----	----	21.7%	75.9%
Lactate ≥ 2 mmol/L	----	----	18.1%	79.5%
COPD	----	----	17.4%	61.3%
CPK ≥ 200 U/L	----	----	13.1%	90.6%
GAP-Diagnosis ≥ 3 days	----	----	----	96.9%
WBC count < 8.5 × 10^3^	----	----	----	93.3%
Male	----	----	----	81.3%
GAP-ICU < 1 day	----	----	----	77.1%

Abbreviations: OR: odds ratio; CI: confidence interval; APACHE II: Acute Physiology and Chronic Health Evaluation; SOFA: sequential organ failure assessment; LDH: lactate dehydrogenase; GAP-ICU: time from diagnosis to ICU admission; GAP-Diagnosis: time from symptoms onset to diagnosis; ICU: intensive care unit; COPD: chronic obstructive pulmonary disease; CPK: creatine phosphokinase; WBC: white blood cells.

## Data Availability

The corresponding author (A.R.) had full access to all study data and assumes responsibility for data integrity and accuracy of analyses. All authors approved the final manuscript. The views expressed are those of the authors and do not necessarily reflect those of SEMICYUC. The data supporting the conclusions are available from the Spanish Society of Critical Care (SEMICYUC) under authorization and are not publicly accessible. Researchers can request the data from the corresponding author (A.R.), with SEMICYUC’s permission.

## Supplementary Materials

The following supporting information can be downloaded at: https://www.mdpi.com/article/10.3390/jcm14155383/s1, Table S1: Performance of multivariate linear model (GLM) for ICU mortality; Table S2: Collinearity study by VIF (variance inflation factors) determination; Table S3: Cross-validation of multivariate linear (GLM) model; Table S4: Performance of balanced linear model; Figure S1: Area under ROC curve (AUC) for multivariate lineal model for ICU mortality; Figure S2: Forest plot with the variables included in the balanced linear model, along with the odds ratio; Figure S3: area under ROC curve of balanced mortality linear model; Figure S4: Category profiles according to the model (A = linear model; B = no linear model).
