# Supplementary material for: Machine Learning-Based Identification of Risk Factors for ICU Mortality in 8902 Critically Ill Patients with Pandemic Viral Infection
Source: J Clin Med. 2025 Jul 30;14(15):5383. doi: 10.3390/jcm14155383 (PMC12346979; doi:10.3390/jcm14155383)
Supplement: Supplementary file 1 [file jcm-14-05383-s001.zip › jcm-3638417-supplementary.pdf]

# Machine Learning-Based Identification of Risk Factors for ICU Mortality in 8,902 Critically Ill Patients with Pandemic Viral Infection

Supplementary material

Table S1: Performance of multivariate linear model (GLM) for ICU mortality.

```
Point estimates and 95% CIs:
--------------------------------------------------------------
Apparent prevalence *                      0.26 (0.24, 0.27)
True prevalence *                          0.11 (0.10, 0.12)
Sensitivity *                              0.61 (0.55, 0.66)
Specificity *                              0.79 (0.77, 0.80)
Positive predictive value *                0.26 (0.23, 0.29)
Negative predictive value *                0.94 (0.93, 0.95)
Positive likelihood ratio                  2.83 (2.51, 3.19)
Negative likelihood ratio                  0.50 (0.43, 0.58)
False T+ proportion for true D- *          0.21 (0.20, 0.23)
False T- proportion for true D+ *          0.39 (0.34, 0.45)
False T+ proportion for T+ *               0.74 (0.71, 0.77)
False T- proportion for T- *               0.06 (0.05, 0.07)
Correctly classified proportion *          0.77 (0.75, 0.78)
--------------------------------------------------------------
* Exact CIs
```

Figure S1:  Area under ROC curve (AUC) for multivariate lineal model for ICU mortality

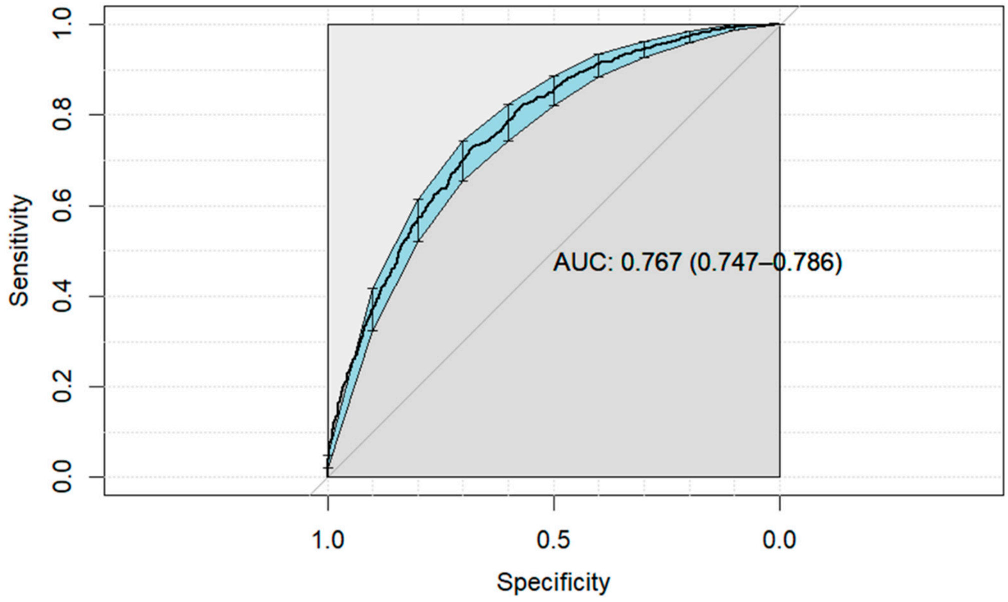

Table S2: Colinearity study by VIF (variance inflation factors) determination. For each variable the VIF number shoud be lower than 5. No colinearity was observed between teh variables included in the model.

(Cut: cut-off; AB: antibiòtics; CPK: creatine phospokinase; DD: D dímer; MR_SA: methicillin-resistant *S. Aureus*; MV: invasive mechanical ventilation; WBC: White blood cells; COPD: chronic obstructive pulmonary disease; dis: disfunction; Chr_Card_dis; chronic cardiac disease; HIV: Human immunodeficiency virus; AKI: acute kidney injury; CRP:C-reactive protein; GAP_ICU_cut: time elapsed between diagnosing pandemic viral infection and admission to ICU; Chr_renal_dis: Chronic renal disease; ID: immunosuppression ; Rx-cutoff: > 2 fields with infiltrations in chest X-ray; PCT: procalcitonin; MS_SA: Methicillin-sensitive *S. aureus*; GAP_diagnsosis_cut: Time from symptoms onset to diagnosis; hematol_dis: Hematologic disease; LDH: Lactate dehydrogenase)

| | | | | | |
|---|---|---|---|---|---|
| Gender | Age_cut | APACHEII_cut | SOFA_cut | GAP_ICU_cut | GAP_diagnosis_cut |
| 1.244341 | 1.276926 | 1.208923 | 1.226265 | 1.093180 | 1.047728 |
| shock | asthma | COPD | chr_card_dis | chr_renal_dis | hematol_dis |
| 1.356423 | 1.045245 | 1.145751 | 1.120188 | 1.235726 | 1.267477 |
| pregnancy | obesity | diabetes | HIV | ID | steroids |
| 1.194002 | 1.074920 | 1.166280 | 1.038319 | 1.280052 | 1.120085 |
| AB_admission | MV_admission | miocardial_dis | AKI | Rx_cutoff | LDH_cut |
| 1.057822 | 1.334363 | 1.045954 | 1.494582 | 1.094705 | 1.207255 |
| CPK_cut | WBC_cut | Creatinine_cut | CRP_cut | PCT_cut | lactate_cut |
| 1.264428 | 1.059907 | 1.390359 | 1.376125 | 1.979526 | 1.536026 |
| DD_cut | klebsiella | Acinetobacter | S.pneumoniae | MS_SA | E.coli |
| 1.633889 | 1.014681 | 1.011345 | 1.062170 | 1.025214 | 1.009540 |
| MR_SA | Pseudomonas | aspergillus | antiviral_vaccine | | |
| 1.013241 | 1.019359 | 1.017844 | 1.072473 | | |

Table S3: Cross-validation of multivariate linear (GLM) model.

```
Confusion Matrix and Statistics

          Reference
Prediction    0    1
         0 1866  509
         1  116  179

               Accuracy : 0.7659
                 95% CI : (0.7494, 0.7819)
    No Information Rate : 0.7423
    P-Value [Acc > NIR] : 0.00263

                  Kappa : 0.2479

 Mcnemar's Test P-Value : < 2e-16

            Sensitivity : 0.9415
            Specificity : 0.2602
         Pos Pred Value : 0.7857
         Neg Pred Value : 0.6068
             Prevalence : 0.7423
         Detection Rate : 0.6989
   Detection Prevalence : 0.8895
      Balanced Accuracy : 0.6008

       'Positive' Class : 0
```

## Development of the GLM linear model for mortality with class imbalance correction

Applying the ROSE package to the training set reduced the population from 6232 patients to 3152 patients. Of these, 1606 died, giving an estimated mortality rate of 50.9%, twice the real rate (25%).

The variables included in the balanced model were the same as those used in the class imbalance model: Male, age cut-off, APACHEII cut-off, SOFA cut-off, ICU GAP cut-off, GAP diagnosis cut-off, shock, asthma, COPD, chronic heart disease, chronic kidney disease, haematological disease, pregnancy, obesity, diabetes, HIV, immunosuppression, steroids, antibiotic treatment on ICU admission, mechanical ventilation on ICU admission, Myocardial dysfunction, acute kidney injury (AKI), > 2 areas of infiltration on chest X-ray, lactate dehydrogenase cut-off, creatine phosphokinase cut-off, leukocyte cut-off, CRP cut-off, PCT cut-off, lactate cut-off, D-dimer cut-off, *Klebsiella* spp, *Acinetobacter* spp, *S. pneumoniae*, methicillin-resistant *Staphylococcus aureus*, methicillin-sensitive *Staphylococcus aureus* (MSSA), *E. coli*, methicillin-resistant *Staphylococcus aureus* (MRSA), *Pseudomonas aeruginosa, Aspergillus* spp and antiviral vaccine.

In Figure S2, the variables included in the models can be seen with their respective Odd Ratios and confidence intervals. The variables independently associated with mortality were the same as those observed in the unbalanced model.

Figure S2: Forest-Plot with the variables included in the balanced linear model with Odds Ratio.

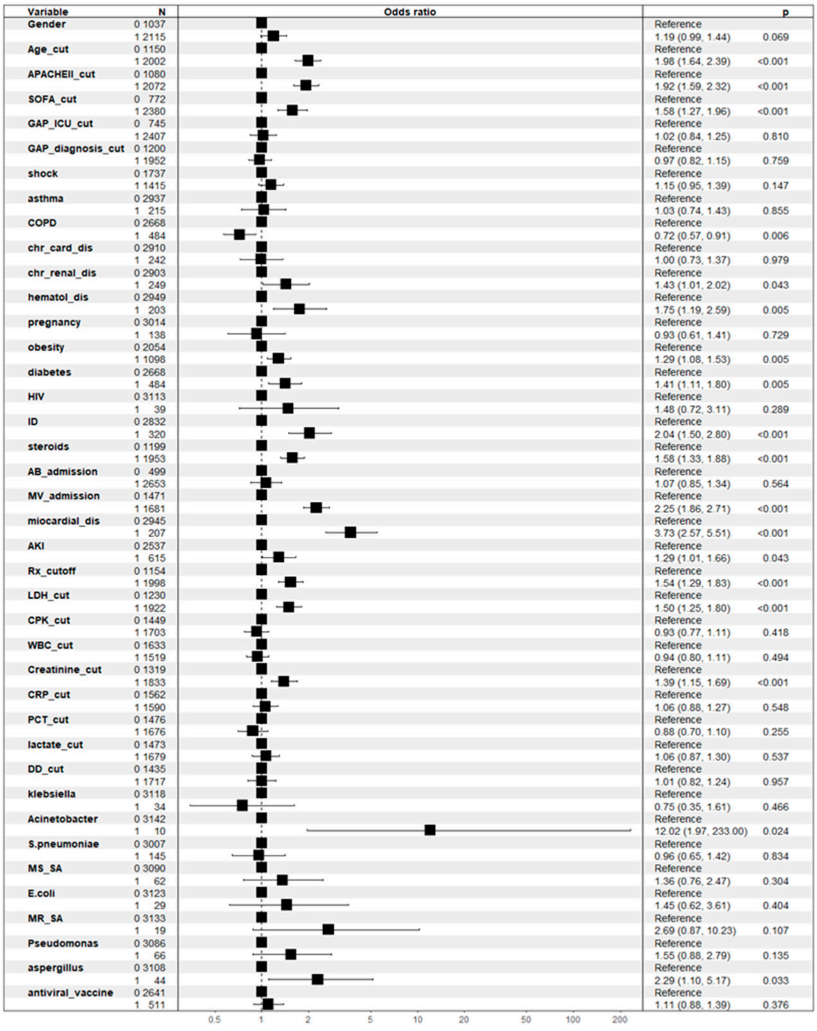

Table S4 : Performance of balanced linear model

Point estimates and 95% CIs:

| | |
|---|---|
| Apparent prevalence * | 0.26 (0.24, 0.27) |
| True prevalence * | 0.43 (0.41, 0.45) |
| Sensitivity * | 0.43 (0.40, 0.46) |
| Specificity * | 0.87 (0.85, 0.89) |
| Positive predictive value * | 0.72 (0.68, 0.75) |
| Negative predictive value * | 0.67 (0.65, 0.69) |
| Positive likelihood ratio | 3.39 (2.93, 3.93) |
| Negative likelihood ratio | 0.65 (0.62, 0.69) |
| False T+ proportion for true D- * | 0.13 (0.11, 0.15) |
| False T- proportion for true D+ * | 0.57 (0.54, 0.60) |
| False T+ proportion for T+ * | 0.28 (0.25, 0.32) |
| False T- proportion for T- * | 0.33 (0.31, 0.35) |
| Correctly classified proportion * | 0.68 (0.67, 0.70) |

* Exact CIs

Figure S3: area under ROC curve of balanced mortality linear model

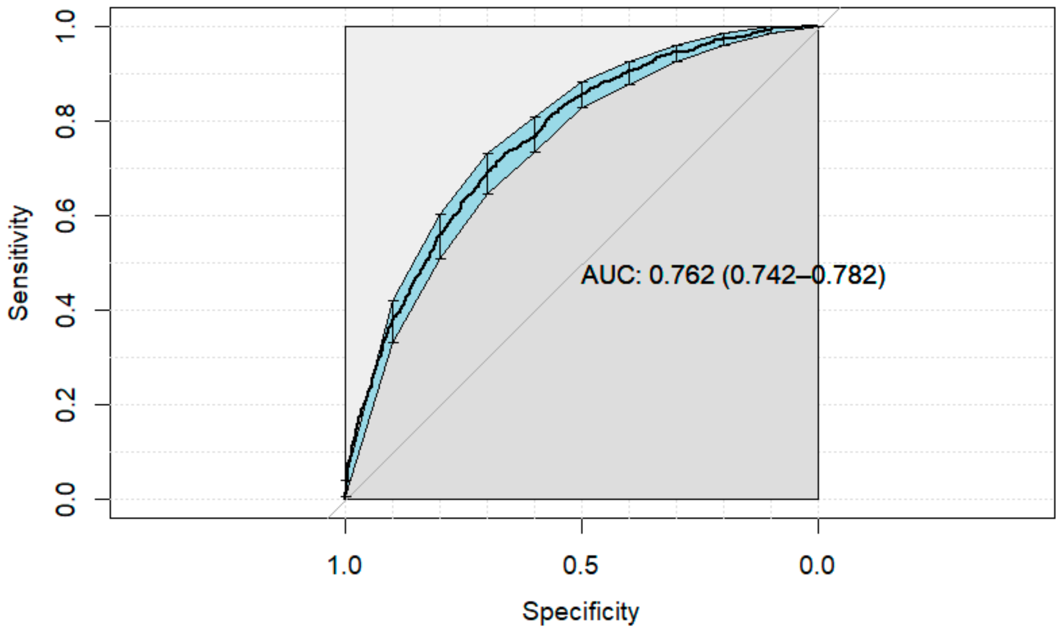

Figure S4: Categories profiles according to the model. A = linear model , B= no linear model

A

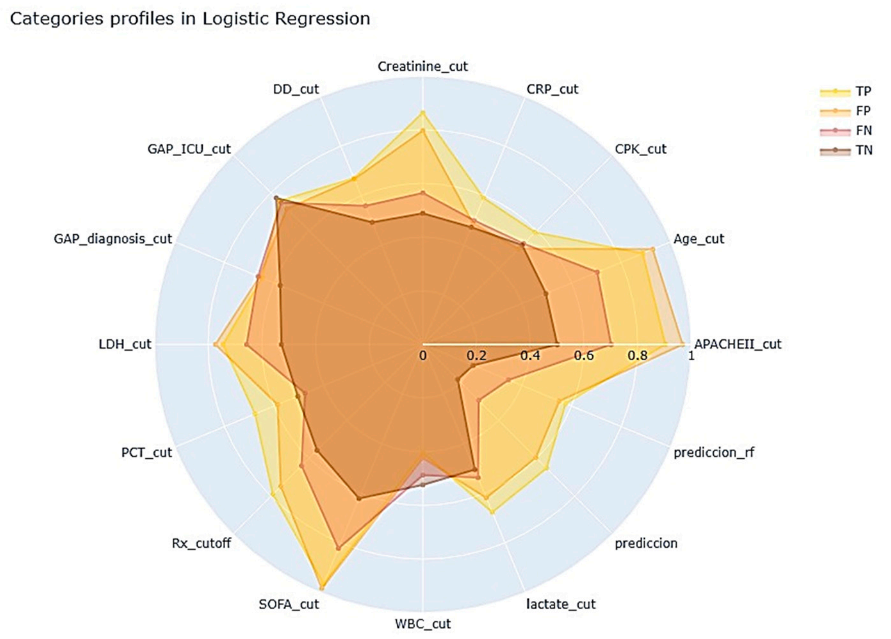


B

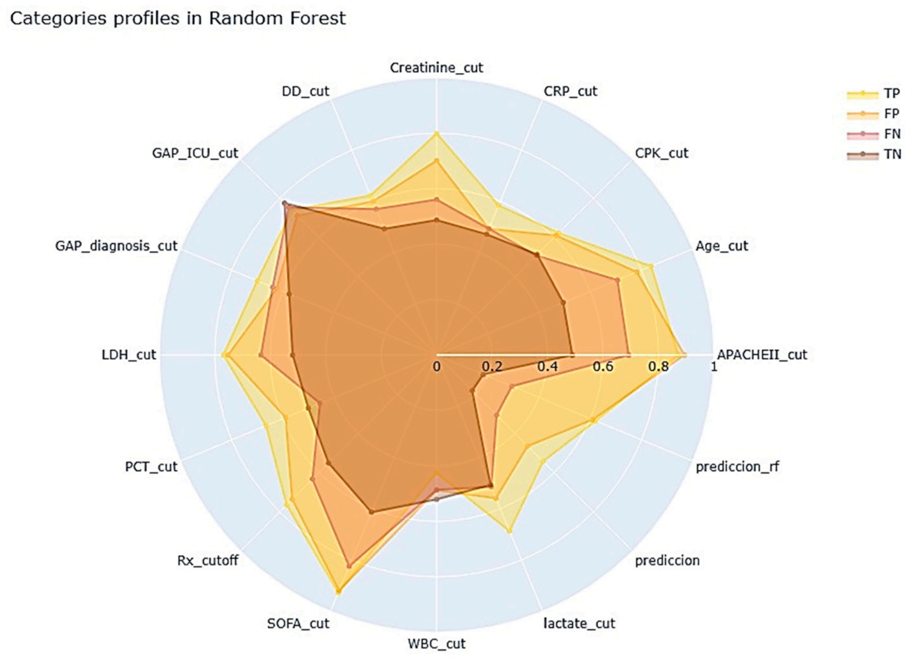


Abbreviations : cut: cut-off; APACHE II: Acute Physiology and Chronic Health Evaluation; SOFA: Sequential organ failure assessment; AB: antibiòtics; CPK: creatine phospokinase; DD: D dímer; MR_SA: methicillin-resistant S. Aureus; MV: invasive mechanical ventilation; WBC: White blood cells; COPD: chronic obstructive pulmonary disease; dis: disfunction; Chr_Card_dis; chronic cardiac disease; HIV: Human immunodeficiency virus; AKI: acute kidney injury;

CRP:C-reactive protein; GAP_ICU_cut: time elapsed between diagnosing pandemic viral infection and admission to ICU; Chr_renal_dis: Chronic renal disease; ID: immunosuppression ; Rx-cutoff: > 2 fields with infiltrations in chest X-ray; PCT: procalcitonin; MS_SA: Methicillin-sensitive S. aureus; GAP_diagnsosis_cut: Time from symptoms onset to diagnosis; hematol_dis: Hematologic disease; LDH: Lactate dehydrogenase)
